# Two-stage surgical management for chronic volar lunate dislocation: a case report

**DOI:** 10.3389/fsurg.2025.1700107

**Published:** 2026-01-08

**Authors:** Lin Zhang, Yang Yu, Fuyin Yang, Jiaze Peng, Jinglin Li, Xianpeng Huang, Xuan Deng, Xuxu Yang, Lidan Yang

**Affiliations:** Department of Orthopedics, Affiliated Hospital of Zunyi Medical University, Zunyi, China

**Keywords:** two-stage surgery, volar lunate dislocation, chronic injury, case report, distraction

## Abstract

**Background:**

Volar lunate dislocations typically result from high-energy trauma involving dorsiflexion and ulnar deviation forces, and are frequently accompanied by peri-lunate fractures. Without timely intervention, these injuries often progress to a chronic stage, conventionally defined as presentation beyond 6 weeks. Conventional single-stage surgical approaches often yield suboptimal outcomes for such complex, chronic presentations.

**Case presentation:**

We report a 34-year-old male with persistent right wrist pain and restricted mobility for 11 months following a fall. Diagnostic imaging confirmed severe chronic volar lunate dislocation. A two-stage surgical protocol—comprising preoperative progressive wrist distraction followed by open reduction, internal fixation, and ligament reconstruction—successfully restored carpal alignment while preserving a functional range of motion. At one-year follow-up, the patient demonstrated excellent clinical recovery.

**Conclusion:**

This case demonstrates that for severe chronic volar lunate dislocations of exceptionally long duration, a two-stage strategy incorporating progressive distraction and subsequent reconstruction is a viable and effective approach. It successfully addressed the challenges of extreme chronicity, achieving anatomical reduction and functional recovery where standard methods might fail.

## Background

Volar lunate dislocations typically result from high-energy trauma, frequently accompanied by peri-lunate fractures. This injury occurs when extreme dorsiflexion and ulnar deviation forces sequentially rupture peri-lunate ligaments, allowing the surrounding carpal bones to displace dorsally while compressing the lunate volarly out of the radial fossa. Delayed diagnosis or inadequate initial management often leads to chronic dislocation, conventionally defined as presentation beyond 6 weeks (1). Therapeutic obstacles include extensive soft tissue contractures, fibrotic scarring, articular stiffness, and secondary osseous and cartilaginous changes, rendering anatomical reduction exceptionally difficult (2). Primary open reduction carries substantial risks of neurovascular injury and frequently fails to maintain stability (3), while untreated cases may progress to scaphoid or lunate avascular necrosis, carpal instability, post-traumatic arthritis, and functional impairment (4, 5). Previously reported cases predominantly had injury durations of 3–7 months in lunate dislocation. The present case, with an injury-to-surgery interval of 11 months, represents a level of chronicity that significantly exceeds most cases described in the existing literature. This extreme chronicity is associated with more severe soft tissue contractures and degenerative changes, rendering reduction and stabilization exceptionally challenging. While a two-stage surgical strategy has been sporadically reported, its application in cases of such prolonged duration remains scarcely explored. Therefore, this case report aims to detail the successful application of a two-stage protocol in this highly challenging scenario. We present the technical details and outcomes to illustrate the protocol's potential as an effective management option for complex, long-standing volar lunate dislocations, thereby expanding the known indications for this technique.

## Case presentation

### Preoperative assessment

**Figure 1:**
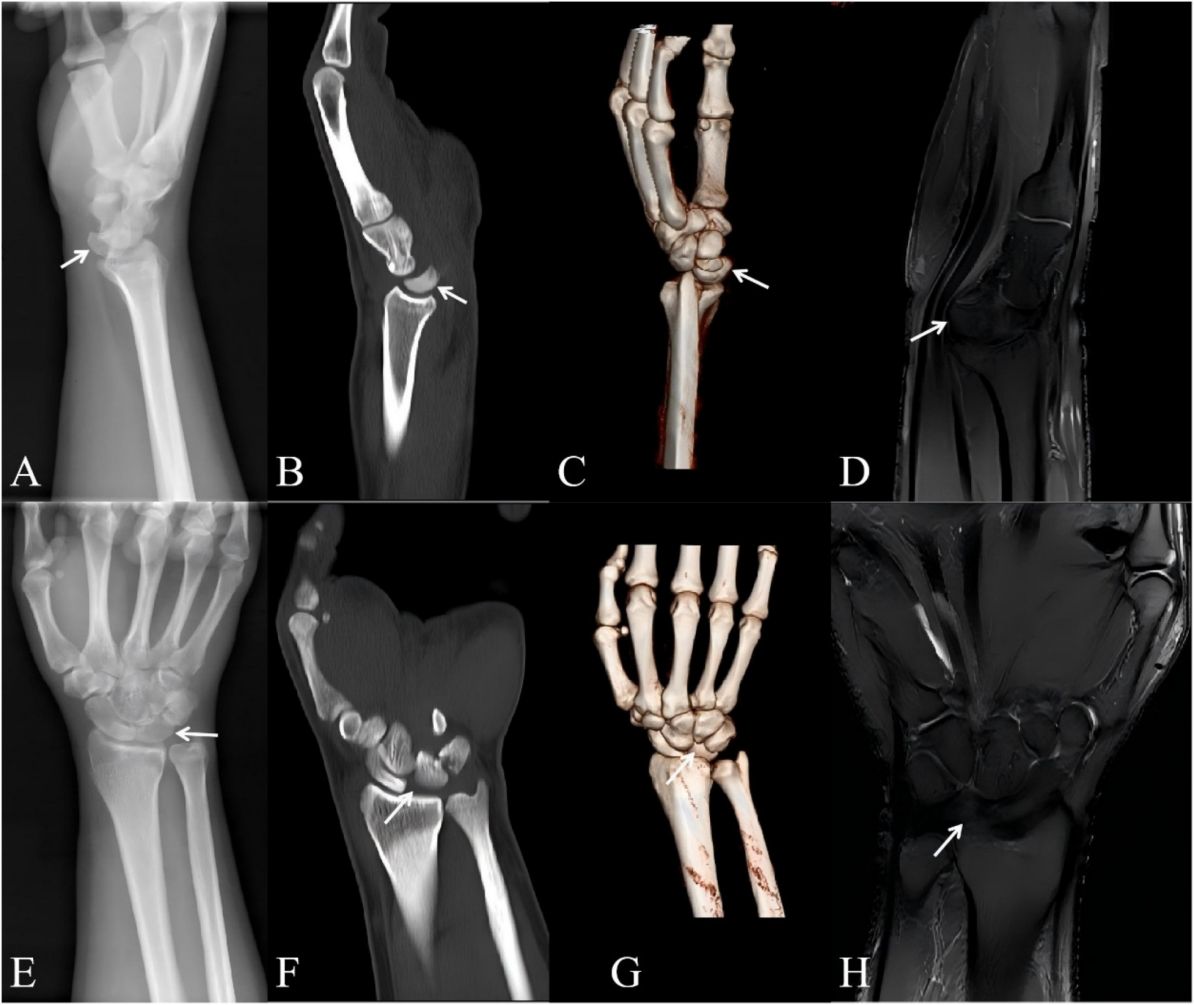
Preoperative imaging: x-ray: lateral radiograph **(A)** and posteroanterior radiograph **(E)**; CT scan: sagittal view **(B)** and coronal view **(F)**; 3D reconstruction: lateral view **(C)** and posteroanterior view **(G)**; MRI: sagittal view **(D)** and coronal view **(H)** of the right wrist. White arrows indicate the volarly dislocated lunate.

A 34-year-old male presented to our institution with persistent right wrist pain and restricted mobility, having sustained a fall from approximately 2 meters in height 11 months prior. The injury occurred during wrist dorsiflexion with initial impact on the palmar surface. After conservative management involving external bracing at multiple institutions failed to alleviate his symptoms, the patient sought definitive care at our facility due to unremitting pain and functional impairment. Physical examination revealed diffuse tenderness with severely restricted active and passive motion. Neurological examination revealed no sensory or motor deficits in the median nerve distribution. Neurovascular status was intact. Imaging (Figures 1A–H) demonstrated volar lunate dislocation, triquetral fracture, and complete rupture of the dorsal radiolunate ligament. The diagnoses were: 1) Chronic volar lunate dislocation; 2) Healed old triquetral fracture; 3) Dorsal radiolunate ligament disruption.

### Intraoperative details

**Figure 2:**
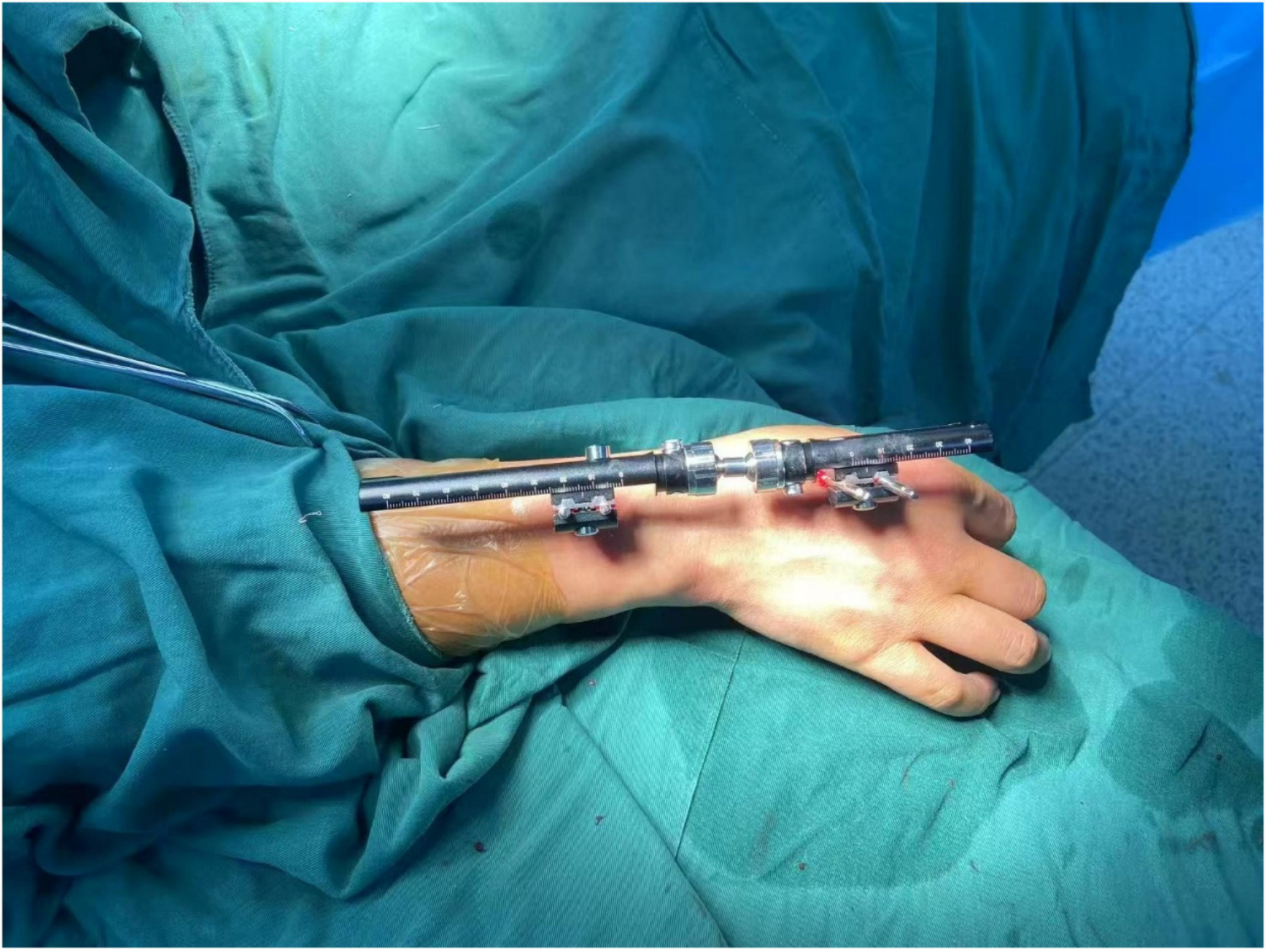
Intraoperative fixator application during stage 1.

**Figure 3:**
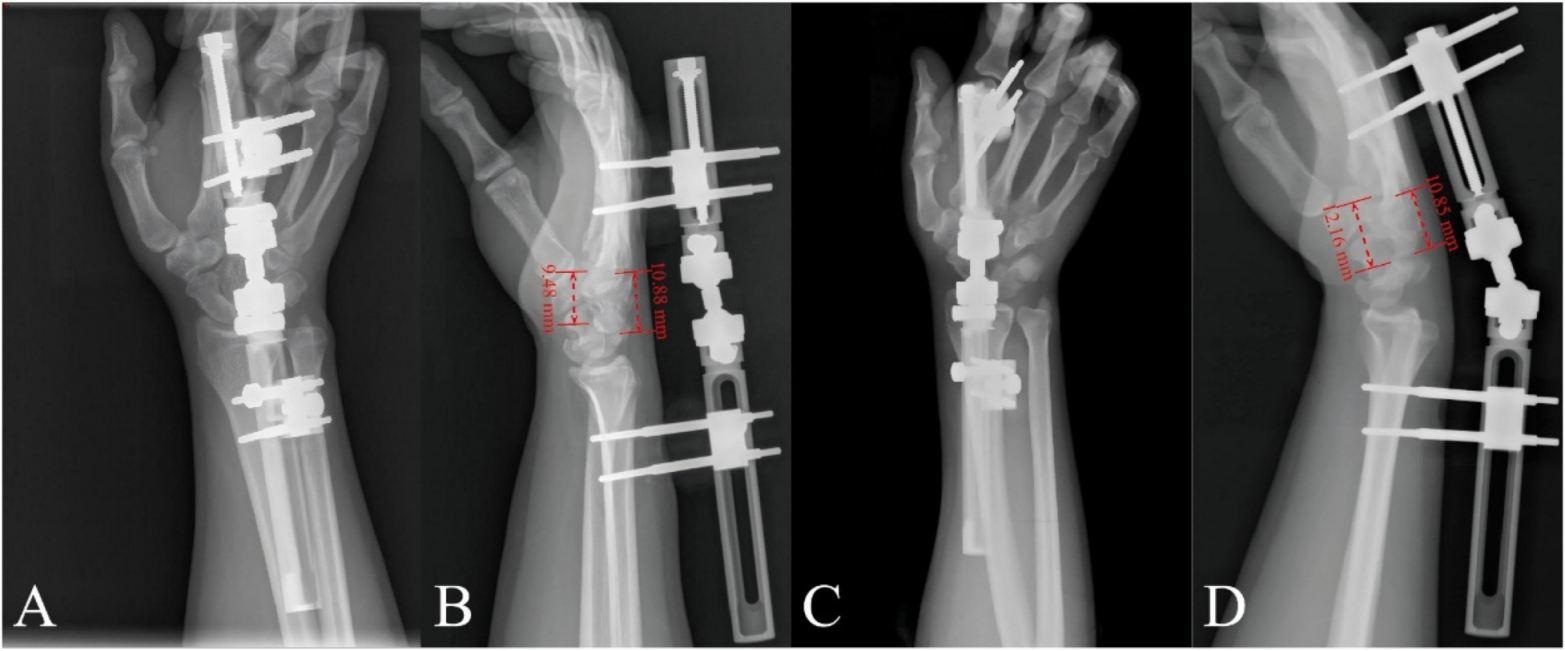
Posteroanterior radiograph **(A)** and lateral view **(B)** during stage 1. posteroanterior radiograph **(C)** and lateral view **(D)** after 3 weeks of distraction. The measured distance in red represents the lunate–metacarpal distance and capitate height.

In Stage 1 surgery, a unilateral, radial-based external fixator (Brand: Orthofix) was applied. Two external fixation pins with a diameter of 4.0 mm were percutaneously inserted into the midshaft of the second metacarpal and distal radius of the right wrist. A unilateral wrist distraction frame was subsequently assembled (Figures 2, 3A,B), maintaining the wrist in neutral extension. Postoperative progressive distraction was initiated at a controlled rate of 1–3 mm/day to achieve gradual elongation of the contracted soft tissues, partial carpal reduction, and creation of adequate space for definitive reduction. Serial radiographic evaluations of lunate-capitate alignment were performed at 3-day intervals. After 3 weeks of distraction, lateral radiographs confirmed achievement of the critical radiographic endpoint: the lunate-second metacarpal distance approximated the height of the capitate (Figures 3C,D). The distraction apparatus was subsequently removed.

Stage 2 surgery was performed immediately through a dorsal wrist approach, exposing the capitate, scaphoid, and volarly displaced lunate. Intraoperative assessment revealed preserved articular cartilage on the capitate, with a suspected grade II chondral lesion (6) (approximately 2 mm in diameter) localized to the dorsoulnar aspect (fissures or defects on the cartilage surface, not involving the full thickness). Scapholunate dissociation and complete lunocapitate dislocation were observed. The lunate exhibited volar angulation (approximately 20°), translational displacement (approximately 8 mm), and supinatory rotation (approximately 30°). Closed reduction attempts proved unsuccessful. Following the release of the lunate's dorsal ligamentous attachments, persistent instability prevented anatomical reduction. Meticulous 360° debridement of the capitate-radius-lunate, scapholunate, and triquetrum-lunate interfaces was performed while preserving volar capsular integrity. The volar capsular attachments at the capitate were subsequently released using a sharp periosteal elevator. Under fluoroscopic guidance, cartilage-protecting reduction instruments facilitated near-complete anatomical restoration. Five 2.8 mm metallic suture anchors (Brand: Arthrex) were then implanted at critical ligamentous insertion sites: two at the lunate footprint, and one each at the capitate, scaphoid, and distal radius. Residual subluxation (approximately 25%–33%) was corrected through strategic tensioning of the anchor sutures. The dorsal radiolunate ligament was augmented using suture anchors, with two placed at the lunate footprint and one at the distal radius, without employing any other grafts. Intraoperative fluoroscopy confirmed satisfactory carpal alignment with preserved articular kinematics. Postoperatively, the right wrist was immobilized in a short-arm cast for three weeks (7).

### Postoperative management

**Figure 4:**
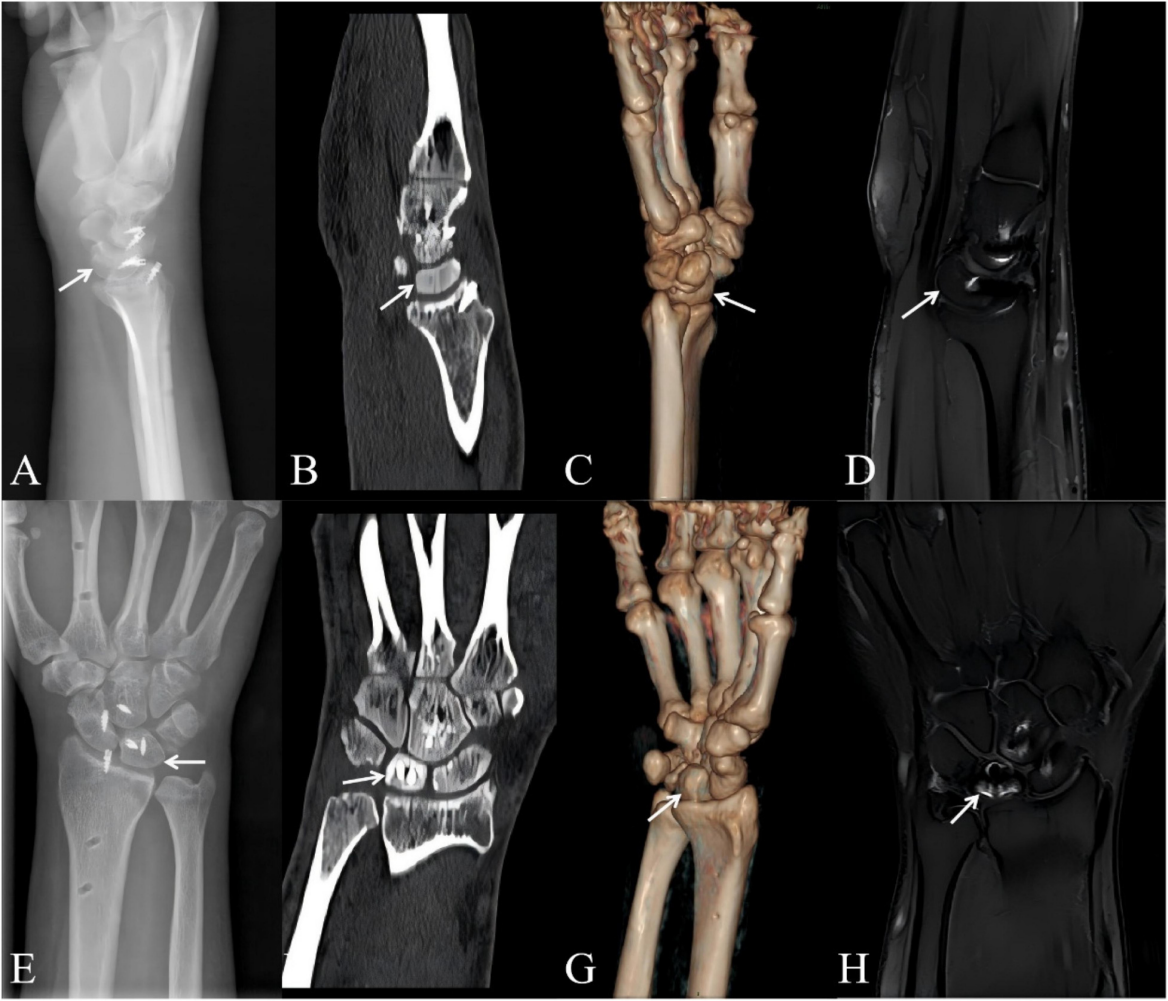
Postoperative imaging: lateral radiograph **(A)** and posteroanterior radiograph **(E)**; CT scan: sagittal view **(B)** and coronal view **(F)**; 3D reconstruction: lateral view **(C)** and posteroanterior view **(G)**; MRI: sagittal view **(D)** and coronal view **(H)** of the right wrist. White arrows indicate lunate reduction.

**Figure 5:**
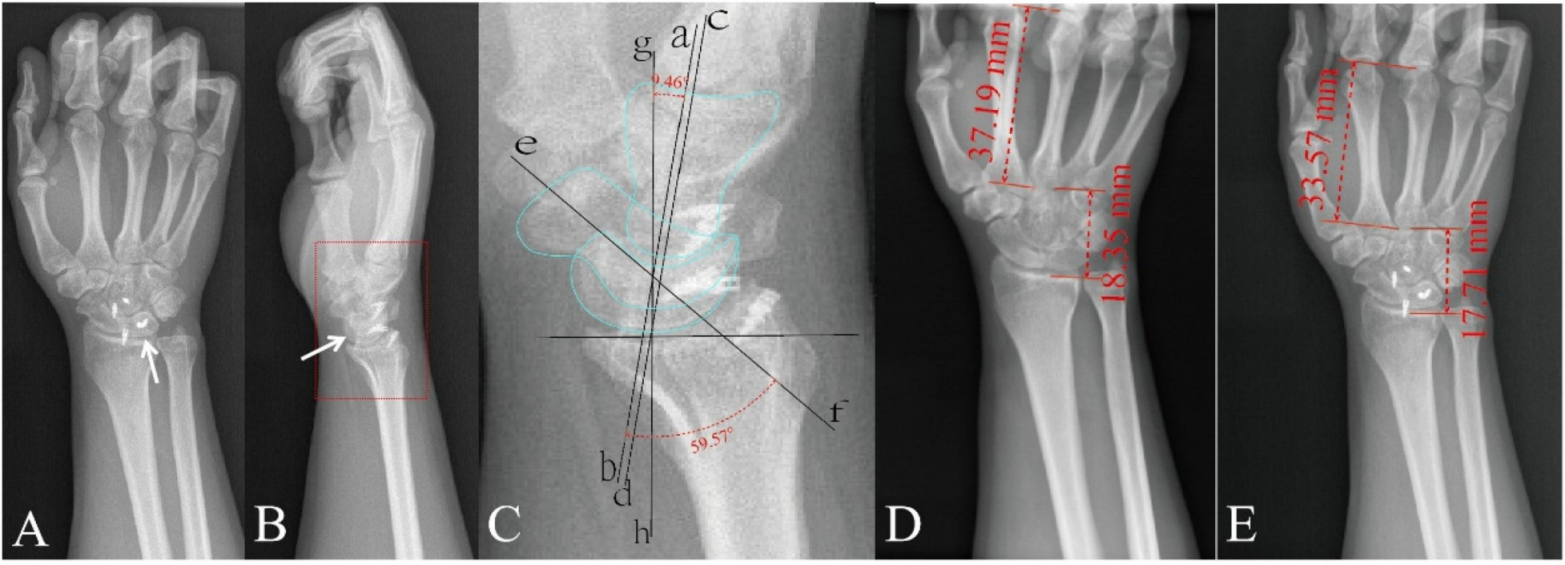
Postoperative one-year follow-up radiographs: posteroanterior radiograph **(A)** and lateral radiograph **(B, C)** is a magnified view of the red box in **(B)**, showing detailed anatomy. Preoperative posteroanterior radiograph **(D)** for comparison. Postoperative posteroanterior radiograph **(E)** White arrows indicate lunate reduction. The measured distance marked in red represents the carpal height and the third metacarpal length. Carpal Height Ratio = Carpal Height/Third Metacarpal Length. Radiolunate angle: 9.46° (angle formed by lunate axis ab and radius axis gh). Capitolunate angle: 0° (angle formed by capitate axis cd and lunate axis ab). Scapholunate angle: 59.57° (angle formed by scaphoid axis ef and lunate axis ab).

**Figure 6:**
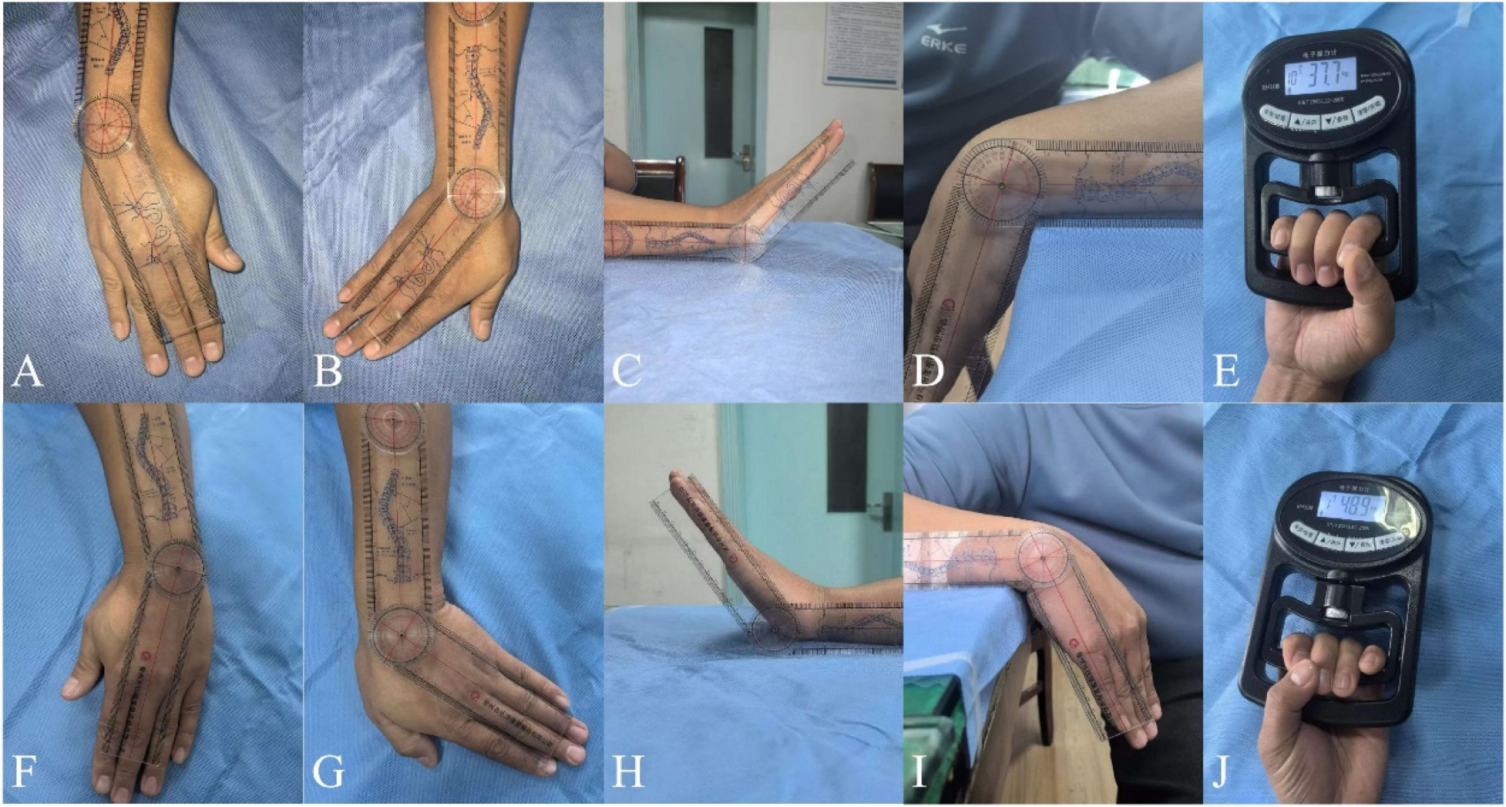
Functional wrist images at 1-year follow-up**:** affected side: radial deviation **(A)**, ulnar deviation **(B)**, dorsiflexion **(C)**, palmar flexion **(D)**, grip strength **(E)**; contralateral side: radial deviation **(F)**, ulnar deviation **(G)**, dorsiflexion **(H)**, palmar flexion **(I)**, grip strength **(J)**.

Active range-of-motion exercises commenced at 6 weeks, progressing to strengthening by 6–12 weeks. Serial imaging confirmed maintained reduction and fracture union (Figures 4A–H). At 12 months, the patient resumed weight-bearing activities with near-complete functional recovery. Outcome scores are summarized in Table 1. Imaging outcomes at 1 year are illustrated in Figures 5A–E. Functional outcomes at 1 year are illustrated in Figures 6A–J.

**Table 1:** Radiographic parameters and clinical outcomes of the patient at one year postoperatively.

Follow-up items	Radiographic parameters	Clinical outcomes
Radiolunate angle(degree)	9.46	—
Capitolunate angle(degree)	0	—
Scapholunate angle(degree)	59.57	—
Carpal height ratio	Baseline: 0.49 Final: 0.53	—
Grip strength (as % of contralateral side)	—	77%
VAS(points)	—	Baseline: 4 Final: 0
Mayo(points)	—	Baseline: 60 Final: 90
ROM(degree)	—	Palmar flexion: 70° Dorsiflexion: 45° Radial deviation: 20° Ulnar deviation: 30°

## Discussion

The surgical management of chronic lunate dislocations typically includes open reduction and internal fixation (ORIF), proximal row carpectomy, wrist arthrodesis, scaphoid excision with four-corner arthrodesis, and arthroscopic reduction. Among these, open reduction and internal fixation is the mainstream surgical approach. However, due to the reduced space between the distal radius and the base of the metacarpals, accompanied by extensive soft tissue contracture, open reduction poses significant challenges and often requires excessive force for relocation, which may damage the articular cartilage of the lunate and the proximal pole of the capitate. Furthermore, restoring anatomical alignment necessitates extensive soft tissue dissection, which increases the risk of avascular necrosis and nonunion of the scaphoid and lunate. Attempting forceful reduction within this constrained space may exacerbate pre-existing cartilage damage on the proximal capitate. Other surgical techniques, to varying degrees, reconstruct wrist stability at the expense of joint mobility (Table 2).

**Table 2:** Surgical options for chronic lunate dislocations.

Technique	Indications	Advantages	Limitations	Refs
ORIF	Failed closed reduction; Instability post-reduction; Absent AVN/repairable soft tissues	Anatomical restoration; Motion preservation	Extensive dissection; Risk of AVN progression; Infection/neurovascular injury	(11–13)
Proximal row carpectomy	Lunate AVN (Stage IV); Advanced arthritis; Low-demand elderly	Reliable pain relief; Partial mobility	Grip weakness; Progressive radiocarpal degeneration	(14–17)
Four-corner fusion	SLAC/SNAC wrist; Scaphoid nonunion	Durable stability; Maintained grip	Restricted motion; Adjacent joint arthritis	(18, 19)
Arthroscopic reduction	Partial ligament tears; Intact cartilage	Minimally invasive; Rapid recovery	Technically demanding; Limited stability	(20, 21)
Total wrist arthrodesis	Pan-carpal arthritis; Structural failure; Failed prior surgeries	Definitive pain control; Stability	Complete motion loss; Compensatory strain	(19, 22)

The two-stage surgical procedure is an underutilized approach, whose key advantage lies in its first-stage gradual traction that progressively stretches the contracted soft tissues and partially reduces the carpal bones prior to surgery. This process minimizes the need for extensive soft tissue dissection and reduces the risk of cartilage damage during the operative procedure, thereby decreasing the incidence of postoperative complications such as skin necrosis, infection, and nonunion. Garg et al. reported a two-stage reduction treatment for trans-scaphoid perilunate fracture dislocations in 16 cases with injury durations ranging from 3.5 to 7 months. All 16 patients were treated with a unilateral wrist distractor during the first-stage traction. Follow-up results showed an average Mayo wrist score of 78, with 9 cases achieving excellent outcomes. Two patients developed reflex sympathetic dystrophy, resulting in fair outcomes (8). Lal et al. (2) employed bilateral single-plane wrist traction for lunate reduction in a case of perilunate fracture-dislocation with a 3-month injury history, ultimately achieving a favorable functional outcome. Similarly, Dhal et al. (3) reviewed 9 cases of chronic perilunate dislocations with an average injury duration of 3.6 months; among them, 5 were treated with bilateral single-plane wrist traction and 4 with unilateral traction, with improved functional outcomes observed in the bilateral traction group.

In the present case, the injury duration was 11 months, significantly exceeding that reported in any previous literature. This may be attributed to a missed diagnosis at the initial treating hospital. Such an extended period likely resulted in soft tissue contracture and scarring within the wrist joint, secondary osseous and cartilaginous alterations, and considerable difficulty in achieving stability, rendering direct closed or open reduction nearly impossible. The failure of the initial manual reduction and release in this case corroborates this challenge. Therefore, progressive traction prior to open reduction and internal fixation was essential to gradually elongate the contracted soft tissues, thereby minimizing intraoperative soft tissue dissection and cartilage damage, and creating crucial reduction space for the second-stage procedure. Furthermore, long-standing dislocation leads to degenerative changes in the articular cartilage and adaptive morphological alterations in the bones (9), as well as degradation of ligament quality due to original structural failure (e.g., rupture of the dorsal radiolunate ligament) and prolonged disuse (10), making it exceedingly difficult to maintain stability after reduction alone and increasing the risk of redislocation or progression to carpal instability. Accordingly, we also performed ligament reconstruction using suture anchors to enhance wrist stability.

Unlike the methods described in the aforementioned studies, we utilized a unilateral external fixator in the first stage. While bilateral external fixation may offer better biomechanical stability by providing distraction from both the radial and ulnar sides, the scarcity of available data—owing to the rarity of such cases—makes it difficult to definitively conclude which approach is superior. Conversely, while a bilateral fixator can theoretically provide more symmetrical distraction across the radiocarpal joint, the critical pathology requiring reduction—the volarly displaced lunate—is primarily situated within the radial portion of the wrist joint, articulating with the radius and capitate. The primary goal of the distraction phase is to regain space in the radiocarpal compartment to facilitate the subsequent reduction of the lunate. In addition, prolonged traction time increases the risk of complications such as over-distraction or pin tract infection due to potential mismanagement. From this perspective, a unilateral external fixator, with fewer pin sites, may be more suitable.

Overall, the two-stage surgical strategy offers a viable solution for extremely chronic and complex volar dislocations of the lunate. However, close monitoring during the traction phase is essential to prevent over-distraction and pin site infections. Although this protocol involves an extended treatment duration, higher costs, and demands strong patient compliance, its unique value in achieving reduction and stability significantly mitigates the long-term burden of complications such as post- traumatic arthritis and allows for maximal functional recovery. Future studies should focus on the long-term outcomes, optimization of indications, and cost-effectiveness analysis of this technique in the management of similarly complex chronic wrist injuries.

## Conclusion

Preoperative progressive distraction mitigates soft tissue contractures, facilitating safer anatomical reduction during subsequent ORIF and ligament reconstruction. For complex, long-standing volar lunate dislocations, this two-stage approach represents a viable and effective solution.

## Data Availability

The original contributions presented in the study are included in the article/Supplementary Material, further inquiries can be directed to the corresponding author.
